# Construction of *rec*B-*rec*D genetic fusion and functional analysis of RecBDC fusion enzyme in *Escherichia coli*

**DOI:** 10.1186/1471-2091-9-27

**Published:** 2008-10-10

**Authors:** Oytun Portakal, Pakize Doğan

**Affiliations:** 1 Biochemistry Department, Hacettepe University Medical School, 06100 Sihhiye, Ankara, Turkey

## Abstract

**Background:**

*recD*, located between *rec*B and *arg*A, encodes the smallest polypeptide (60 kDa) of the heterotrimeric enzyme RecBCD in *Escherichia coli*. RecD is a 5'-3' helicase and is required for the nuclease activity of RecBCD and for tight binding to dsDNA ends. Here, we have tested the hypothesis that RecD regulates the structure and activities of RecBCD, including RecA loading.

**Results:**

To characterize its regulatory functions, *rec*D was genetically fused to *rec*B through deletion and substitution mutations. The *rec*B-*rec*D fusion led to a decreased amount of the heterotrimer. Both fusion mutants proved to be recombination proficient, viable and resistant to DNA damaging agents, and to have DNA unwinding, ATP-dependent dsDNA exonuclease and Chi genetic activities.

**Conclusion:**

Our findings suggest that the *rec*B-*rec*D fusion may form a RecBD fusion protein and therefore affect RecD assembly, but this does not change the three-dimensional structure of the heterotrimer.

## Background

RecBCD is the key enzyme of double strand (ds) breaks repair and recombination in *Escherichia coli*[1-3]. RecBCD enzyme is a large heterotrimer (330 kDa) comprising RecB, RecC and RecD polypeptides [4,5], and has dsDNA and single strand (ss)DNA exonuclease, ssDNA endonuclease, helicase, DNA-dependent ATPase and RecA loading activities [1-3,6]. This multifunctionality enables the enzyme to initiate homologous recombination. RecBCD carries out the presynaptic step of recombination to produce a 3' single-strand overhang. The E. coli hotspot Chi (5' GCTGGTGG 3') stimulates recombination near to and distant from its 3' end, so it facilitates the enzyme's task [6,7].

Genetic and physical assays show that recombinational DNA repair in E. coli occurs as follows: if there is a ds break in the chromosome, RecBCD specifically binds to the blunt or near-blunt dsDNA ends [8,9]. Unlike other helicases, RecBCD has two independent helicase subunits, RecB and RecD, so the enzyme is a processive helicase (>30 kb) and begins to unwind the dsDNA [10,11]. Initially the enzyme degrades from the 3'-ended strand until it reaches the Chi sequence in excess Mg^+2 ^ions. Thereafter, it degrades the complementary strand as unwinding continues [12]. The combined helicase and nuclease effects produce a 3' overhang [12-14]. RecBCD loads the strand-exchange protein, RecA, on to this 3' overhang to form a RecA-ssDNA filament. This filament catalyzes the formation of a D-loop with a homologous dsDNA, which develops a Holliday junction structure. Postsynaptic resolution produces recombinants [15].

A key aspect of this process is the interaction between Chi and RecBCD, which regulates the structure and activities of the enzyme. Chi is a recombinational hotspot in the RecBCD pathway and stimulates recombination up to 30-fold [16,17]. During unwinding, this octameric sequence is recognized by the RecC subunit of the enzyme [18-20], if RecBCD only approaches from the right side [8], and Chi and enzyme then interact [21]. The enzyme continues to unwind after Chi, though more slowly [22], and at the end of the DNA the subunits disassemble to form an inactive enzyme [23]. During the continued unwinding, the nuclease activity of the enzyme degrades the 5'-end strand [12], and the enzyme begins to load RecA on to the 3'-end strand to the left of Chi [24]. The mechanisms of this regulation are not yet well understood, but it has been suggested that RecD plays a role [25]. On the basis of similarities between the features of RecBCD lacking RecD and RecBCD interacting with Chi, the following hypothesis has been proposed: RecD is ejected at Chi to form a recombinogenic enzyme [26,27]. However, subsequent studies have shown that purified RecBC is different from the enzyme interacting with Chi: RecBC lacks exonuclease and Chi recognition activities, but can load RecA constitutively [20,28,29]. Recent single-molecule studies have indicated that RecD remains associated with the RecBC subunits at the Chi site [30,31]. On the other hand, RecD inhibits RecA loading activity before the Chi site; a point mutation in the RecA loading domain of RecB eliminates RecA loading activity, but removing RecD from these mutants reverses this inactivation [32,33]. Against the hypothesis that RecD is eliminated at the Chi site, we propose that RecD is required to regulate RecA loading activity. To test this proposal, we generated two *rec*B-*rec*D genetic fusions that would allow us to examine the regulatory functions of RecD. We tested four predictions: [1] the fusion enzymes cannot bind specifically to dsDNA ends and lack all enzyme activities; [2] the fusion enzymes can bind to the ends but cannot unwind the dsDNA; [3] the fusion enzymes can bind and unwind dsDNA, but lack nuclease and Chi genetic activities; [4] the fusion enzymes behave like wild type.

In this study we describe the *rec*B-*rec*D fusion mutants that have wild type phenotypes. Both fusion enzymes are recombination proficient, can repair DNA damage, and have DNA unwinding, exonuclease and Chi genetic activities. However, the fusion between *rec*D and *rec*B reduced the heterotrimer level. Our findings suggest that *rec*B-recD fusion may form RecBD, a fusion protein, and may therefore affect assembly, but this effect does not change the three-dimensional structure of the heterotrimer.

## Results

### *rec*B-*rec*D fusions were generated by recombineering

**Figure 1:**
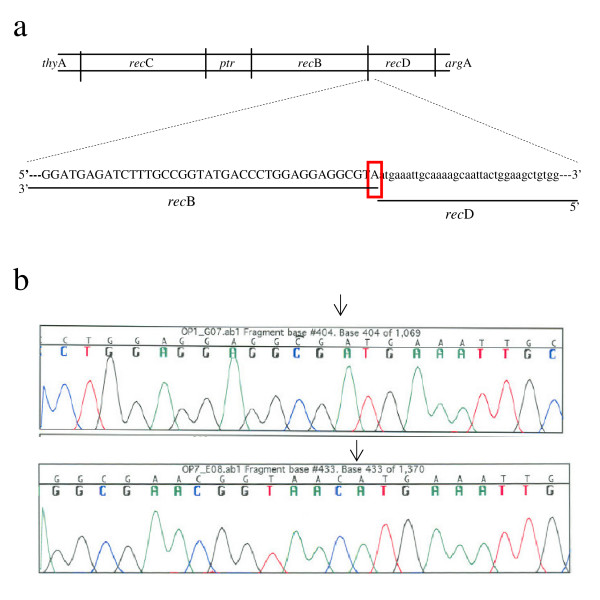
**a**.** The physical map of the *thy*A-*arg*A region of the E. coli K-12 chromosome, and the nucleotide sequence of the *rec*B-*rec*D overlap site where *rec*BD fusion mutations were constructed.** The nucleotide A that overlaps the stop codon *rec*B (TAA) and the start codon *rec*D (atg) is shown in the square. *rec*B nucleotides are depicted in capital letters while *rec*D nucleotides are depicted in small letters in order to distinguish them. **b**. The sequence analysis results of the deletion (top) and substitution (bottom) fusion mutations after mutagenesis by recombineering.

RecBCD is composed of the products of *rec*B, *rec*C and *rec*D, which lie between the *arg*A and *thy*A intergenic regions of E. coli (Figure 1a). *ptr*, which encodes protease III, is located between *rec*C and *rec*B. The *rec*B termination codon overlaps the initiation codon of the downstream *rec*D by one nucleotide. Therefore, *rec*B and *rec*D form an operon with a major external promoter for both genes and with a minor internal promoter for *rec*D. The major promoter to the left of *rec*B directs the transcription of the 3543 and 1824 nucleotides of the *rec*B and *rec*D coding sequences, respectively, whereas the weaker promoter to the left of *rec*D appears to direct the transcription of *rec*D alone. To generate a genetic fusion between *rec*B and *rec*D, the mutations were targeted to this overlap site. Two fusion forms were generated: [1] a deletion fusion mutation and [2] a substitution fusion mutation. For the former, two nucleotides (TA) of the *rec*B termination codon at the overlap site were deleted; this removes the termination codon of *rec*B but does not yield a frameshift mutation. For the latter, three codons encoding two asparagine and one glycine residues, which has the potential of forming equivalent hydrogen bonds and facilitates a beta turn respectively, were substituted at the same site. This substitution leads to a short bridge between *rec*B and *rec*D.

Recombineering is the name given to the method that produces recombinants by the Red-recombination function of lambda phage. Short linear homologous DNA strands (e.g. 20–70 bases) are required. Recombination can occur between single-stranded or double-stranded DNAs [34] by the exo, beta and gamma proteins of lambda. Single-strand pairing is formed between a synthetic oligonucleotide containing a mutation and single-stranded chromosomal DNA arising from replication fork. This requires beta, an annealing protein, and a 35-nucleotide flanking homology on each end. To increase the frequency of recombination, the oligonucleotide should correspond to the lagging strand at the replication fork and the direction of replication should be counter-clockwise [35]. In this study, the deletion and substitution fusion mutations were generated by single-strand pairing. Appropriate oligonucleotides were created and synthesized, then transformed into an E. coli strain containing lambda prophage (Table 1). Chromosomal DNAs isolated from the transformed cells were then amplified with appropriate primers. Six candidates with DNAs expected to encode RecBD fusion proteins were found for both mutants by colony-screening, and two were isolated by transduction (Figure 1b). They were named V2825 and V2826.

**Table 1:** *Escherichia coli* strains and plasmids used in this study

strains	genotype	source, ref,
HME63	Δ(argF-lac) U169 λ cI857 Δcro-bioA galK tyr 145 UAG mutS<>amp	Court, 2003
AFT325	thyA argA21 recF143 hisG4 met rpsL31 galK2 xyl-5 λ^- ^F^-^	Amundsen,86
V2825	hisG4 met rpsL31 galK2 xyl-5 *rec*BD2728	This study
V2826	hisG4 met rpsL31 galK2 xyl-5 *rec*BD2729	This study
V2831	hisG4 met rpsL31 galK2 xyl-5 *rec*BCD2731 (*rec*BCDΔ)	This study
V66	argA21 hisG4 recF143 met rpsL31 galK2 xyl-5 λ^- ^F^-^	Schultz,83
V67	RecB21::IS186 argA21 hisG4 recF143 met rpsL31 galK2 xyl-5 λ^- ^F^-^	Schultz,83
V222	argA::Tn10 argA21 hisG4 recF143 met rpsL31 galK2 xyl-5 λ^- ^F^-^	Amundsen,90
V1306	Thi-1 relA1 λ^- ^(Hfr PO44)	Schultz,83
594	Lac-3350 galK2 galT22 rpsL179 λ^- ^F^-^	Schultz,83
C600	Thr-1 leuB6 thi-1 lacY1 tonA21 supE44 rfbD1 λ^- ^F^-^	Schultz,83

plasmids		

pBR322χ +F225	-	Smith, 81
pDWS2	*rec*BCD in pBR322	Ponticelli,85
pBD2728	2ntΔ*rec*BD fusion in pBR322	This study
pBD2729	9nti *rec*BD fusion in pBR322	This study

### Haploid alleles of *rec*B-*rec*D fusion mutants have wild type phenotype

Purified transductants were subjected to the phage spot test on LB plate with λ red^-^gam^-^χ^+^, λ red^-^gam^-^χ^0^, λ red^+^gam^+^, T4, T4 gene 2^- ^P1 and P2 phages (Table 2). The sizes of plaques were evaluated [17] (Table 3): [1] λ red^-^gam^-^χ^0 ^formed small plaques, λ red^-^gam^-^χ^+ ^formed medium plaques, and λ red^+^gam^+ ^formed large plaques. In *rec*BCD^+ ^cells, λ red^-^gam^-^χ^0 ^forms small plaques and λ red^-^gam^-^χ^+^forms large ones, whereas in *rec*BC mutants, χ^0 ^and χ^+ ^phages forms plaques of the same size. Our result indicates that V2825 and V2826 have recombination-proficient phenotypes. [2] There were many plaques in the P1 spot. P1 lysogeny requires RecBCD recombination function, so fusion mutants are recombination-proficient [3]. λ red^-^gam^-^χ^+^produced larger plaques than λ red^-^gam^-^χ^0^; this result suggests that there is a Chi hotspot activity. [4] T4 formed plaques, but T4 lacking gene 2 protein did not. Gene 2 protein protects T4 DNA ends from RecBCD exonuclease degradation. In the absence of gene 2, T4 cannot form plaques. This result indicates that the *rec*B-*rec*D fusion mutants have exonuclease activity. [5] Many plaques were found in the P2 spot. P2 phage growth requires the exonuclease activity of RecBCD. Our result suggests that fusion mutants have exonuclease activity.

**Table 2:** Phage strains used in this study

Phage*	Genotype	Source, ref.
1081	λ sus J6 b1453 cI857 χ^+ ^D123	F. and M. Stahl
1082	λ b1453 χ^+ ^D123 sus R5	F. and M. Stahl
1083	λ sus J6 b1453 χ^+ ^76 cI857	F. and M. Stahl
1084	λ b1453 χ^+ ^76 sus R5	F. and M. Stahl
T4	T4B	E.B. Goldberg
T4 gene2^-^	T4 gene 2 amN51	E.B. Goldberg
P1	P1 vir-1	G.R. Smith
P2	P2 vir-1	R. Calender
801	λ^+^	G.R. Smith

*:Phages 1081–1084 obtained from G.R. Smith collection. b1453 is a deletion mutation of lambda removing *int, red *and part of *gam*. The χ^+^76 mutants are derived from a phage containing the b1453 mutation.

**Table 3:** Phage spot test results in haploid alleles of *rec*BD fusion mutants

*rec *alleles*	λ phage			T4 phage		P1 phage	P2 phage
	λ red^-^ gam^-^χ^0^	λ red^-^ gam^-^χ^+^	λ red^+ ^ gam^+^	gene 2 pr(-)	gene 2 pr(+)		
*rec*BCD	small	medium	large	+	-	+	+
*rec*BD2728	small	medium	large	+	-	+	+
*rec*BD2729	small	medium	large	+	-	+	+

* Strains are transformants of V2831 (Δ*rec*BCD2731) with the indicated *rec *alleles on the chromosome.

### *rec*B-*rec*D fusion mutants are recombination-proficient

The recombination proficiency of haploid alleles in the *rec*B-*rec*D fusion mutants was measured in lambda vegetative crosses [16,17]. Table 4 shows the proficiency of recombination by each mutant with *rec*BCD^+^, *rec*B null mutant and the *rec*D mutant. Both the deletion and substitution fusion mutants gave high recombination proficiencies, similar to that of *rec*BCD^+ ^cells. *rec*B null mutants were found to be recombination-deficient and *rec*D mutants were recombination-proficient, as expected.

**Table 4:** Lambda hotspot cross results in haploid alleles of *rec*BD fusion mutants

*rec *alleles*	λ Recombination Frequency (%J+R+)^#^	Chi Activity^#^
*rec*BCD	7.48 ± 1.86	6.03 ± 0.78
*rec*B21	0.13 ± 0.08	1.06 ± 0.09
*rec*D1013	6.33 ± 1.24	1.23 ± 0.16
*rec*BD2728	5.82 ± 0.83	5.20 ± 0.28
*rec*BD2729	7.5 ± 1.25	6.69 ± 0.88

* Strains are transformants of V2831 (Δ*rec*BCD2731) with the indicated *rec *alleles on the chromosome except *rec*B21 and *rec*D1013 which are derivatives of V66 (*rpsL*31).

### *rec*B-*rec*D fusion mutants have Chi activity

Chi activity was measured in hotspot crosses to determine whether the fusion mutants interact with Chi [16,17] (Table 4). The ratio of frequency of recombination in a genetic interval with or without a Chi site gives Chi hotspot activity. Both deletion and substitution fusion mutants produced high Chi hotspot activities similar to that of wild-type cells (approximately 6.0), indicating that more exchange occurs in an interval with a Chi site than that an interval without one. *rec*BCD null mutants and *rec*D mutants produced no Chi hotspot activity (approximately 1.0).

### *rec*B-*rec*D fusion mutants on plasmids have wild type phenotypes

The phenotypes of the *rec*B-*rec*D fusion mutants on plasmids were tested by spot tests [17] (Table 5). λ red^-^gam^-^χ^+^produced larger plaques than λ red^-^gam^-^χ^0^. T4 formed plaques, but T4 gene 2^- ^did not. P1 and P2 produced plaques. It was concluded that *rec*B-*rec*D fusion mutants on plasmids are recombination-proficient and they have Chi activity and exonuclease activity.

**Table 5:** Phage spot test results of *rec*BD fusion mutants on plasmid

*rec *alleles*	λ phage			T4 phage		P1 phage	P2 phage
	λ red^-^ gam^-^χ^0^	λ red^-^ gam^-^χ^+^	λ red^+ ^ gam^+^	gene 2 pr(-)	gene 2 pr(+)		
+	small	medium	Large	+	-	+	+
*rec*BD2728	small	medium	large	+	-	+	+
*rec*BD2729	small	medium	large	+	-	+	+
-	small	small	large	+	+	-	-

*Strains are transformants of strain V2831(Δ*rec*BCD2731) with derivatives of plasmid pBR322 containing the indicated *rec *alleles. "-" means pBR322.

### *rec*B-*rec*D fusion mutants on plasmids are recombination-proficient

The recombination proficiency of *rec*B-*rec*D fusion mutants on plasmids was tested by conjugational cross [17] (Table 6). His^+ ^recombinants in the plasmid derivatives of *rec*BD2728 and *rec*BD2729 fusion mutants increased to the wild-type value. These results show that the fusion mutants are Rec^+^.

**Table 6:** Hfr conjugation results of *rec*BD fusion mutants on plasmid

*rec *alleles*	Hfr conjugation (%His + [Str+])^#^
*+*	1.13 ± 0.057
*rec*BD2728	1.26 ± 0.17
*rec*BD2729	1.31 ± 0.13
-	0.00

*: Strains are transformants of strain V2831(Δ*rec*BCD2731) with. the indicated *rec *alleles on derivatives of plasmid pBR322. "-" contains pBR322.

^#^: Results are the mean ± SEM from 2 and 3 experiments.

### There are RecBD fusion polypeptides

**Figure 2:**
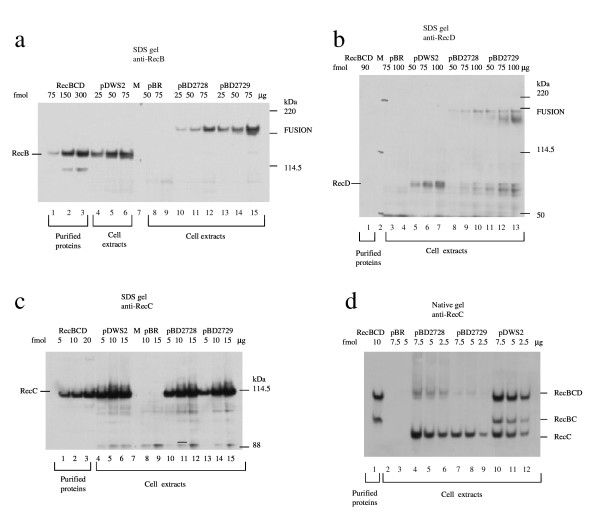
**RecBD fusion polypeptides are shown in lanes 10–15 of part a and in lanes 8–13 of part b.** Intact RecC polypeptides are shown in lanes 10–15 of part c and in lanes 4–9 of part d. RecBD fusion mutants do not assemble into the heterotrimer (in lanes 4–9 of part d). Extracts were from late log phase cultures of V2831 containing derivatives of plasmids pDWS2 (*rec*BCD^+^), pBD2728 and pBD2729. Fusion polypeptides in cell extracts were separated on denaturing (3–8% Tris acetate or 4–12% Bis-Tris buffer) and native (5% in 2 mM EDTA, 50 mM MOPS pH 7.0) polyacrylamide gels. Proteins were transferred to PVDF membrane and probed with mouse monoclonal antibodies specific for RecB, RecC and RecD, which were captured and visualized with horseradish peroxidase-linked antimouse IgG.

We wanted to determine whether the construction of a *rec*B-*rec*D fusion leads to an active RecBD fusion polypeptide. For this purpose, we performed native and denaturing polyacrylamide gel electrophoresis of crude extracts and blotted them. RecBD fusion polypeptides were detected on SDS-PAGE probed with anti-RecB and anti-RecD (Figures 2a, b), and RecC polypeptide was detected when the gels were probed with anti-RecC (Figures 2c, d). In the native gel, less heterotrimer was found in the two fusion mutants than in purified RecBCD, but neither intact RecB polypeptide nor RecBC was detected in cell extracts of either fusion mutant (Figure 2d). Unexpectedly, a polypeptide with size similar to RecD was observed (Figure 2b).

### RecBDC fusion enzymes have ATP-dependent exonuclease activity

Because the fusion peptides were found on gels and they were recombination-proficient, we wondered whether the fusion mutants have enzyme activities. We first tested exonuclease activity in them. RecBCD degrades linear DNA to acid-soluble nucleotides by two activities, dsDNA and ssDNA exonucleases, both of which are ATP-dependent. We measured only ATP-dependent dsDNA exonuclease activity in this work [36]; in both fusion mutants, it was approximately half that in wild type (Table 7). This result is consistent with the qualitative assays for exonuclease activity with T4, T4 gene 2 and P2 (Table 5).

**Table 7:** ATP-dependent dsDNA exonuclease activity results of *rec*BD fusion mutants

*rec *alleles*	dsDNA exonuclease
+	333 ± 37.47
*rec*BD2728	118.60 ± 16.67
*rec*BD2729	126.35 ± 22.30
-	18 ± 2.44

*: Strains are transformants of strain V2831 (*rec*BCDΔ) with derivatives of plasmid pBR322 containing the indicated *rec *alleles. "-" means pBR322

^#^: Results are the mean ± SEM from 3 experiments.

### RecBDC fusion enzymes have DNA unwinding and Chi cutting activities

**Figure 3:**
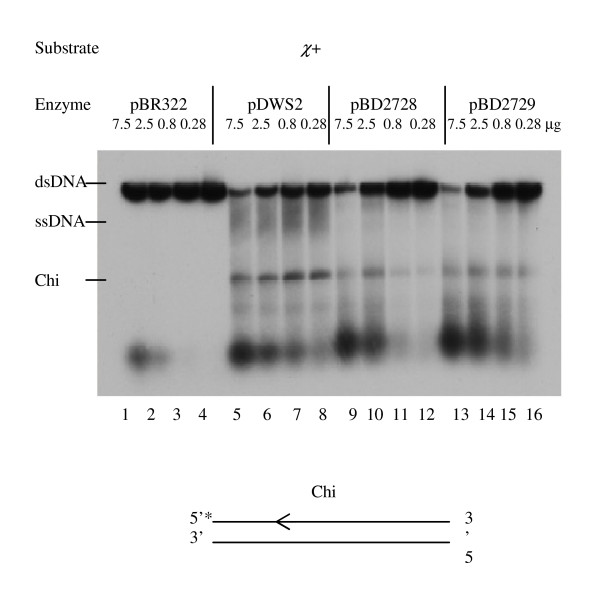
**RecBD fusion enzymes unwind dsDNA and make a cut after Chi.** DNA unwinding and Chi cutting activities of the fusion enzymes were measured using pBR322 χ + DNA substrate labeled with 5'-^32^P. DNA substrates (4.5 nM) were incubated with crude extracts at 3.5 mM magnesium, monitored on 1% agarose gels and analyzed by autoradiography.

Since the fusion enzymes are recombination-proficient and have Chi genetic activity, we wanted to demonstrate DNA unwinding and Chi cutting activities, which are measured together on linear dsDNA labeled with ^32^P at the 5' end of a single strand containing a Chi site [24]. In the presence of excess magnesium, wild-type RecBCD binds to dsDNA ends and degrades the 3'-ended strand until Chi site, so few if any ssDNA fragments are found. After Chi, the enzyme cuts ssDNA a few nucleotides to the 3' side of Chi and forms a Chi fragment (Fig 3, lanes 5–8) [see Additional file 1, lanes 3–5]. When we tested the fusion enzymes for this activity, the deletion fusion mutant enzyme had unwinding and exonuclease activities, so it did not produce a full-length ssDNA fragment but could cut near Chi and form Chi fragments (Figure 3, lanes 9–12). Similarly, the substitution fusion mutant enzyme unwound dsDNA and degraded the 3'-end strand, so it did not form an ssDNA fragment but formed Chi fragments (Figure 3, lanes 13–16). These observations are consistent with the phage test results.

### *rec*B-*rec*D fusion mutants are resistant to DNA damaging agents

**Figure 4:**
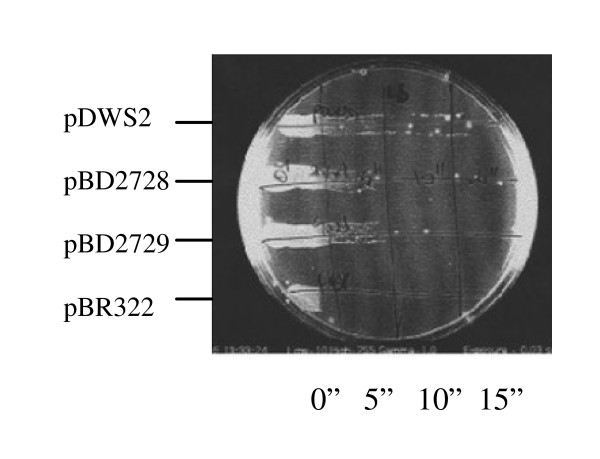
**The survival of *rec*BD fusion strains after exposure to UV is similar to wild type.** Strains are transformants of V2831 containing *rec*BD fusion alleles on a plasmid. Cultures were grown to mid log phase at 37°C in LB broth, exposed to UV radiation for 0, 5, 10 and 15 s, and plated on LB agar. Survival is defined as the fraction of initial colony forming units surviving after UV exposure.

**Figure 5:**
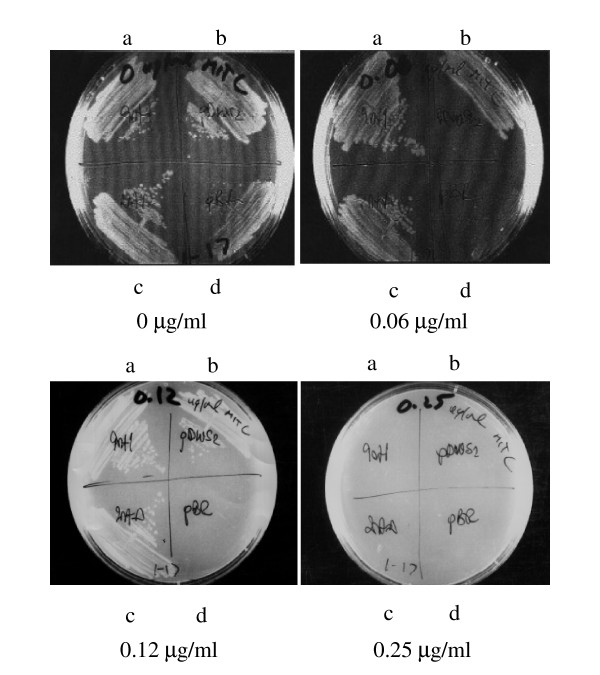
**Both RecBD fusion mutants are resistant to Mitomycin C. **Strains are transformants of V2831 containing rec alleles on a plasmid. Cultures were grown to mid log phase in LB broth, and plated on LB agar plates with or without Mitomycin C. Survival was determined by colony formation. **a, b, c **and **d **represent pBD2729, pDWS2, pBD2728 and pBR322, respectively.

The viability of the fusion mutant cells was tested by their sensitivity to DNA damaging agents [37,38]. Deletion and substitution fusion mutants were just as resistant to UV radiation as the *rec*BCD wild type strain when compared to pBR322 (Figure 4). Both fusion mutants showed similar ability to the *rec*BCD wild type strain in reducing DNA damage induced by Mitomycin C (Figure 5), but plasmids were sensitive. These data show that *rec*B-*rec*D fusions do not inhibit DNA repair.

## Discussion

We set out to characterize the regulatory function of RecD. To our knowledge, this is the first study involving the generation of genetic fusions between two RecBCD subunits. We observed that the fusion enzymes have wild type phenotypes. Below, we discuss possible inferences from our results.

### Fusion enzymes resemble wild type

The phenotypes of the fusion enzymes were demonstrated by genetic and enzymatic assays (Table 8). The deletion fusion enzyme is recombination proficient and resistant to DNA damaging agents (Table 3, 4, 5, 6, Figures 4, 5). In contrast to RecBC, this mutant enzyme has nuclease and Chi genetic activities (Table 7, Figure 3). It can unwind dsDNA as wild type enzyme does and recognizes the Chi sequence because it forms Chi fragments (Figure 4). Similarly, we found that the substitution fusion enzyme is recombination proficient and resistant to DNA damaging agents (Table 3, 4, 5, 6, Figures 4, 5). This mutant enzyme has Chi activity, nuclease and helicase activities, and also recognizes Chi (Table 7, Figure 3). Our findings indicate that both fusion enzymes can bind to dsDNA ends and unwind double strands. During unwinding they can recognize the Chi sequence and form a 3' overhang. After interaction with Chi, wild type RecBCD enzyme loads RecA protein on to the free 3' end [24], which allows pairing and strand exchange with homologues [15], so this activity is required for homologous recombination [33]. We did not measure RecA loading activity in this study, but the genetic measure of recombination proficiency and enzymatic measure of Chi recognition [32] afford evidence for such activity.

**Table 8:** Summary of *rec*BD fusion mutant phenotypes

*rec *alleles*	DNA damaging agents		Recombination			Enzyme Activities		
	Mtc C	UV	%J+R+	Hfr conjugation	Chi activity	dsDNA exonuclease	DNA unwinding	Chi cutting

+	R	R	+	+	+	+	+	+
*rec*BD2728	R	R	+	+	+	+	+	+
*rec*BD2729	R	R	+	+	+	+	+	+
-	S	S	-	-	-	-	-	-

*: Strains are tranformants of V2831(*rec*BCD2731) with the indicated *rec *alleles on derivatives of plasmid pBR322. "-" contains pBR322.

(R, resistant to agent; S, sensitive to agent)

### Fusion enzymes are active

**Figure 6:**
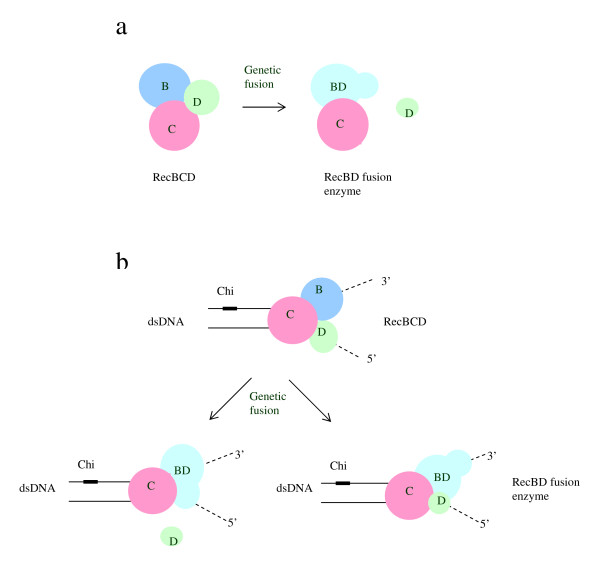
**Model for the structures and functions of RecBDC fusion enzyme.a**. The construction of RecBD fusion enzyme. **b**. RecBD fusion enzyme may assemble with RecC and may form an active fusion heterotrimer, or wild type length RecD may assemble to fusion heterotrimer yielding unusual subunit associations.

In our mutations we removed the stop codon of *rec*B, so translation continued to the end of the *rec*D coding sequence and produced a RecBD fusion heterodimer, which might affect the folding of the enzyme and consequently its functions. Folding is a rapid process initiated by local elements that assemble to yield the final native fold [39,40]. Our protein analysis indicated not only the presence of RecBD fusion polypeptide, as we expected (Figures 2a–c), but also the absence of intact RecB monomer and RecBC dimer (Figure 2d). This result is the most important evidence for a RecBD fusion heterodimer. An intact RecC that we found is essential for assembly and association into holoenzyme. However, we observed that the amount of heterotrimer was lower than in wild type. One possible explanation is that the assembly of the fusion protein to form the heterotrimer was affected because an oligomeric protein is formed by consecutive folding and association of the constituent polypeptides. A more significant finding than the reduction of heterotrimer level is RecD with wild type length. This indicates that the RecD polypeptide is expressed by its minor promoter, which may affect the assembly process. In E. coli, *rec*B *and rec*D form an operon with two promoters; the major promoter is external and directs the transcription of both genes, whereas the minor one is internal and appears to direct the transcription of *rec*D. A similar promoter composition is found in other E. coli operons such as *glnALG *and *rpsU*-*dnaG-rpoD*; the minor promoter allows downstream genes to be transcribed under certain conditions [6]. However, it is not yet clear when the weaker promoter for rec*D *governs, so we did not take it into consideration at the outset. Nevertheless, wild type length RecD could bind to Rec(BD fusion)-RecC assembly and could make Rec(BD fusion)-RecC-RecD enzyme that might be responsible for the functionality (Figure 6).

During our study the three-dimensional structure of RecBCD was discovered. It was found that the distance between 1174 of RecB polypeptide and 2 of RecD polypeptide is about 60.8 Å (Wigley and colleagues, personal communication) [see Additional file 2], which is longer than was thought. This distance corresponds to about 17 amino acids. However, in our study, three amino acids were substituted between 1180 of RecB and 1 of RecD, so it corresponds to total 10 amino acids (about 35 Å). Therefore at least seven more amino acids may be required to fuse *rec*B and *rec*D. Furthermore, this long distance between C-terminal of RecB and N-terminal of RecD may cause a functional enzyme which may be assembled by Rec(BD fusion)-RecC-Rec(BD fusion), in which RecB and RecD come from two different polypeptides.

Genetic studies showed that there is little or no Chi hotspot activity in frameshift or single amino acid mutations of RecC(*rec*C*, *rec*C343 respectively) [17-19]. Therefore it was proposed that hotspot Chi is recognized by RecC as the 3' single strand DNA during unwinding [5,18-20]. It was suggested that this recognition initiates a signal, which causes a change in RecD polypeptide to form 3'overhang to the left of Chi for RecA loading [20]. Based on our observations, it can be suggested that this signaling in both RecBDC fusion enzymes would retain in response to Chi.

The results presented here suggest that RecBD fusion enzymes have wild type phenotype and fully active. However, considering the distance between C-terminal of RecB and N-terminal of RecD in the three-dimensional structure, and the presence of wild type-length RecD, unusual subunit associations such as Rec(BD fusion)-RecC-RecD and Rec(BD fusion)-RecC-Rec(BD fusion) may be formed and may be responsible for the activity. To rule out this possibility, purification of those discrete molecules and then their extensive biochemical assays are required. For this purpose, purification methods, SDS-PAGE analysis and MALDI-TOF MS can be performed [41,42].

## Conclusion

*rec*B-*rec*D genetic fusion in E. coli demonstrates that RecBDC fusion enzymes are recombination proficient and active as well as wild type, so we infer that three-dimensional structure of fusion proteins does not change. The reduced heterotrimer formation may arise from the failure of RecD assembly process. However, wild type length RecD requires a purification to eliminate subunit associations that might be responsible for the enzyme activities.

## Methods

### Bacteria, phage and plasmids

Bacterial strains and plasmids are shown in Table 1, and phages are listed in Table 2 with their genotypes and sources. *rec*BCD^+ ^means *rec*B^+^, *rec*C^+ ^and *rec*D^+^. Rec^+ ^indicates a strain with *rec*BCD^+ ^and *recA*^+^.

### Media

Tryptone broth, minimal medium (OMBG), phage suspension medium (SM) and LB (Lurial bertani) agar have been described [17]. BBL agar contains trypticase (BBL-microbiology systems, Maryland); BBL-YE contains 0.1% yeast extract. Terrific broth contains yeast extract, trypticase and glycerol.

### Materials

Oligonucleotides were purchased from Integrated DNA Technologies Inc (IDT, Coralville, IL, USA), the chromosomal DNA isolation kit from Promega (Madison, WI, USA), the protein assay kit and non-fat dried milk from BioRad (USA), electrophoresis equipment for DNA from Ellard Instrumentation (USA) and for protein from BioRad and Invitrogen (USA); and chemicals were purchased from Sigma Chemical Company (USA) and Fisher Scientific (USA).

### Mutagenesis

For recombineering, two oligonucleotides were designed. The first, containing a TA deletion at the *rec*B stop codon, 5'-TACTCTACAAACGGCCATACTGGGACCTCCTCCGCTACTTTAACGTTTTCGTTAATGACCTTCGACACCT-3', was used for the deletion mutation. The second, containing an aac-ggt-aac substitution at the overlap site, 5'-CCTACTCTACAAACGGCCATACTGGGACCTCCTCCGCTTGCCATTGACTTTAACGTTTTCGTTAATGACCTTCGACACC-3', was used for the substitution mutation. Oligonucleotides corresponded to the lagging strand at the replication fork and contained a 35 nucleotide flanking homology on each side [34,35]. Recombineering was performed on an E. coli strain (HME63) lacking the mismatch repair system but containing lambda prophage. A fresh culture of HME63 at 32°C was divided into two flasks. Half the culture was not induced but was kept for future use. The other half was induced by incubating at 42°C for 15 min, then on ice for 15 min. The culture was centrifuged at 4600 g and the pellet was dispersed in 1 ml cold water; 30 ml cold water was added and the suspension was centrifuged. The pellet was again dispersed in 1 ml cold water and centrifuged, and the pellet was dispersed in 200 μl cold water. Forty-five μl of induced cells was transferred into three electroporation cuvettes. Fifteen pmol of fusion oligo and 15 pmol of gal oligo were added to the second and third cuvettes, respectively. Forty-five μl of uninduced cells was transferred to two electroporation cuvettes. Fifteen pmol of gal oligo was added to the second cuvette. Electroporation was performed at 1.8 kV. LB (1 ml) was added to each cuvette. The cells were diluted with SM and spread on LB plates.

### Mutant detection

A large chromosomal fragment (1.7 kb) containing fusion mutations was amplified. Two primers were used; the forward was relative to 1046 bp to the mutation site, and the reverse was relative to 656 bp (IDT). Transformed cells were grown overnight at 42°C. Fifty patch-grid plates were prepared from these fresh colonies. The cells were lysed at 96°C for 8 min. The chromosomal DNA was amplified in a reaction mixture containing 0.2 μM of each primer, 1.5 mM MgCl_2_, 0.2 mM dNTPs and 1 U Taq polymerase (Invitrogen, San Diago). The conditions of amplification were as follows: 94°C for 15 s, 55°C for 30 s, and 72°C for 2 min (30 cycles). PCR products were digested by BsaXI for the deletion mutation, and by NlaIII for the substitution mutation (New England BioLabs). As a result, approximately 1750 colonies were screened by colony PCR. A total of 5 candidates were detected for the deletion mutation and 1 candidate for the substitution mutation. Those candidates were purified and sequenced (Big Dye Cycle Sequencing Protocol, PE Biosystems).

### Mutant isolation

Mutagenesis was generated in an E. coli strain (*thy*^+^*arg*^+^*rec*BCD^+^) containing lambda prophage. To remove lambda phage, the mutants were transferred to another strain (*thy*^-^*arg*^-Δ ^*rec*BCD) by P1 transduction and then *thy*^+^*arg*^+^recombinants were selected. P1-mediated transduction was performed by the plate stock method [17]. A cell culture grown at 32°C overnight was mixed with P1 phage grown on the donor strain at an MOI of 0.5 and incubated at 37°C for adsorption. The cells were grown overnight, harvested and centrifuged. The supernatant was the P1 phage stock containing the mutant strain. Phages were mixed with fresh E. coli cells (*thy*^-^*arg*^-Δ ^*rec*BCD) at 37°C for adsorption. Sodium citrate was added to stop phage adsorption. Transductants were selected on minimal medium containing histidine and methionine, then purified. The phenotypes of the mutant candidates were tested by a phage sensitivity test on LB plate [17]. Fresh cultures of fusion mutants in TB were mixed with BBL-top agar, and poured on to BBL agar plates. Seven phages (10^4 ^pfu/ml) were placed on the spots. Recombination frequency and Chi genetic activity were tested by λ red^-^gam^-^χ^+^, λ red^-^gam^-^χ^0^, λ red^+^gam^+ ^and P_1 _phages: λ red^-^gam^-^χ^+^and λ red^-^gam^-^χ^0 ^produce plaques in Rec^+^cells. The sizes of the plaques in those spots reflect Chi activity. P1 needs Rec^+ ^cells to grow. Exonuclease activity was tested by T4, T4 gene 2^- ^and P_2 _phages. Gene 2 protein binds to the ends of T4 DNA and protects T4 DNA from the exonuclease activity of RecBCD after injection into the cell. P2 requires RecBCD exonuclease activity to grow. Recombination frequency and Chi genetic activity were assayed quantitatively by hotspot crosses as described by Schultz [17]. First, the stocks of lambda phages 1081, 1082, 1083 and 1084 were prepared. Cross 1 was made between parents 1081(c^+^) and 1082 (cI857), and Cross2 was made between parents 1083(c^+^) and 1084 (cI857). A fusion mutant culture (100 μl) was infected with 5 phages per cell. The mixture was incubated at 37°C for phage adsorption. The total phage count was titered on a (sup^-^) strain on BBL whereas the recombinant phage count was titered on a (sup^+^) strain on BBL-YE plates. Recombination frequency (%J^+^R^+^) was determined by the percentage ratio of recombinant phage count to total phage count on the same interval for two crosses. Chi activity was determined by counting the turbid plaques on a (sup^-^) strain on BBL plates and clear plaques on a (sup^+^) strain on BBL-YE plates. The square root of ratio of the turbid plaques to clear plaques in Cross 1 to Cross 2 gives the Chi activity.

### Cloning of fusion mutants

A 18.3 kb BamH1 fragment containing either wild type *rec*BCD or *rec*B-*rec*D fusion mutants was ligated into the BamHI site of pBR322 by T4 DNA ligase (New England BioLabs), and transformed into V2831 *thy*^-^-*arg*^- ^cells to select *thy*^+^-*arg*^+ ^cells. Transformants were confirmed by sequencing. The orientation of the plasmid was tested by Pst1 digestion.

### Preparation of cell-free extracts and protein assays

Crude cell extracts were prepared by a modified Eichler-Lehman method [36]. Isolated colonies of purified transformants were grown in terrific broth. Fresh culture was grown to OD_650 _>2.0, centrifuged and washed. The pellet was resuspended in 50 mM Tris pH 7.5, 10% sucrose and 1 mM EDTA. Aliquots were stored at -70°C in liquid nitrogen. Cells were thawed, their volume was measured, and 100 mM PMSF, 50 mM DTT and 500 mM EDTA were added to reach the final concentration. Lysozyme (10 mg/ml) was added to the mixture and incubated on ice for 5 min. NaCl (5 M) was added, incubated on ice and centrifuged for 30 min. The supernatant was removed and the protein concentrations of the extracts were determined (BioRad). Native gels (5% polyacrylamide, 37.5:1 acrylamide:bis in 50 mM MOPS-KOH, pH 7.0) and SDS gels (3–8% of polyacrylamide in 50 mM Tris-acetate buffer, pH 8.25, or 4–12% of polyacrylamide in 1.25 M Bis-Tris buffer, pH 6.5–6,8) were poured in gel cassettes [20]. The gels were prerun for 1 h, and samples were mixed with loading solution and run at 100 V overnight at 4°C. Proteins were transferred to PVDF membranes (Immobilon-P, Millipore) and probed with mouse monoclonal antibodies specific for RecB, RecD or RecC. The antibodies were visualized with horseradish peroxidase-linked antimouse IgG and a Phototope-HRP detection kit (New England Biolabs).

### Hfr conjugation

The recombination frequency of the fusion mutants on plasmids was tested by Hfr crosses. The donor V1306 strain (*his*^+ ^*rps*L^+^) was mixed with the recipient *rec*B-*rec*D fusion mutants (*his*G4 *rps*L31) as described by Schultz [17]. The ratio of donor to recipient was 1:10. Mating cells were incubated in a 37°C water-bath for 8 min. Diluted mating cells were grown for 20 min and were plated on selective minimal medium (OMBG) containing methionine and streptomycin to select *His*^+ ^*str*^*R *^recombinants. Diluted recipient cells (10^-5 ^dilutions) were plated on LB+amp. The number of *His *exconjugants per donor corrected by viability of the recipient strains is called the recombination frequency.

### Cell viability assays

The viability of *rec*B-*rec*D fusion mutant cells on plasmids was determined by two qualitative assays. In both, small colony formation means low cell viability, and no growth means sensitivity to that agent. In the UV sensitivity assay, fresh overnight cultures were grown to mid-log phase and exposed to UV (10 erg/m^2^/s) for 5–20 s [37]. Cells were incubated at 37°C in the dark overnight, and colony growth was observed on the following day. In the Mitomycin C(MC) sensitivity assay [38], plasmid alleles of the fusion mutants were streaked on LB agar plates containing 0, 0.06, 0.12, 0.25 or 0.50 μg Mitomycin C, incubated at 37°C overnight, and checked for colony growth.

### Enzyme activity assays

DNA unwinding, Chi cutting and dsDNA exonuclease activities were determined in crude extracts of the fusion mutants. ATP-dependent dsDNA exonuclease activity was measured according to Eichler-Lehman [36]. Crude extracts were mixed with [^3^H] T7DNA, 25 μM ATP, BSA, 500 mM Tris HCl pH 8.5, 100 mM MgCl_2 _and 10 mM DDT and incubated at 37°C for 20 min. Carrier DNA (0.2 mg/ml) and 5% TCA were added and the mixture was incubated on ice and centrifuged. The supernatant, containing acid-soluble nucleotides, was counted using a Beckman Coulter Scintillation Counter. One unit of enzyme was defined an amount that solubilizes 5 nmol of dsDNA at 37°C in 20 min.

Chi cutting and DNA unwinding activities were measured together [24]. The substrate, plasmid DNA χ^+^F, was made linear with HindIII (New England BioLabs), incubated with shrimp alkaline phosphatase (SAP) (Invitrogen) at 37°C for 30 min, and labeled with γ^32^P at the 5'ends by T4 polynucleotide kinase (Invitrogen) at 25°C for 30 min. Free nucleotides were removed with an SR-200 mini column. DNA substrate (4.5 nM) was mixed with the indicated amount of cell-free extract in 20 mM MOPS pH 7.5, 3.5 mM MgCl_2_, 5 mM ATP, 1 mM DTT, and 1.6 μM SSB (Promega). After 2 min, ATP-γS and M13 DNA were added. Reaction products were separated on a 1% agarose gel and analyzed by autoradiography.

## Abbreviations

λ: lambda phage; θ: θ replication; χ: Chi; BBL: Bacto tryptone medium; BBL-YE: Bacto tryptone-yeast extract medium; BSA: bovine serum albumin; dsDNA: double strand DNA; DSB: double strand break; gal oligo: oligo containing suppressor mutant; HFR: high frequency recombination; HME63: E.coli cells containing lambda prophage; LB: Luria bertani medium; OMBG: minimal medium; P1: phage P1; P2: phage P2; P22: phage 22; SAP: shrimp alkaline phosphatase; SM: suspension medium; ssDNA: single strand DNA; T4: phage T4; T4 gene 2^-^: phage T4 does not contain gene2 protein; TB: tryptone broth; *thy-arg*: *thy*A-*rec*C-*ptr-rec*B-recD-*arg*A genetic site in E.coli map; UV: ultraviolet.

## Authors' contributions

OP carried out genetic studies, activity assays, statistical analysis, and drafted manuscript. PD participated in the coordination of the study. All authors read and approved the final manuscript.

## Supplementary Material

Additional File 1 *rec*BD fusion mutants have DNA unwinding and Chi cutting activity. The data provided the Chi fragments of *rec*BD fusion mutants with purified RecBCD. Detection of dsDNA unwinding and Chi cutting activities of the RecBD fusion mutants in crude extracts. Extracts were prepared from strain V2831 with the plasmid carrying the indicated *rec *alleles. DNA substrate was derived from pBR322 χ^+ ^and labeled with ^32^P at the 5'end as described in Materials and Methods. Substrate (4.5 fmol/assay) was incubated with indicated amount of crude extracts and purified RecBCD at 37°C for 2 min in the reaction mixture containing 3.5 mM MgCl_2 _and 5 mM ATP. The reaction was stopped by addition of EDTA (0.125 M) and SDS (2.5%). Lanes 1 and 2 show the initial dsDNA substrate and the heat-denatured substrate, respectively. The reaction products were monitored on a 1% agarose gel and analyzed by autoradiography. Click here for file

Additional File 2 The distance between C-terminal of RecB and N-terminal of RecD polypeptides. This picture clearly shows the distance between C-terminal of RecB and N-terminal of RecD polypeptides in 3D structure of RecBCD. Click here for file
