# Supplementary figures and images for: Construction of recB-recD genetic fusion and functional analysis of RecBDC fusion enzyme in Escherichia coli
Source: BMC Biochem. 2008 Oct 10;9:27. doi: 10.1186/1471-2091-9-27 (PMC2586629; doi:10.1186/1471-2091-9-27)

Substrate

$\chi^+$

| Enzyme | - | - | RecBCD |      |      | pBD2728 |     |     |     | pBD2729 |     |     |     |      |         |
|--------|---|---|--------|------|------|---------|-----|-----|-----|---------|-----|-----|-----|------|---------|
| Boil   | - | + | 0.5    | 0.25 | 0.12 | nM      | 7.5 | 2.5 | 0.8 | 0.28    | 7.5 | 2.5 | 0.8 | 0.28 | $\mu$ g |

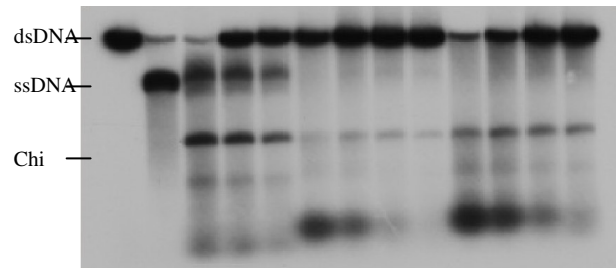

1 2 3 4 5 6 7 8 9 10 11 12 13

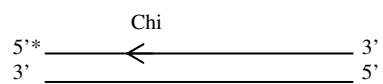

Supplement: Additional File 1 — recBD fusion mutants have DNA unwinding and Chi cutting activity. The data provided the Chi fragments of recBD fusion mutants with purified RecBCD. Detection of dsDNA unwinding and Chi cutting activities of the RecBD fusion mutants in crude extracts. Extracts were prepared from strain V2831 with the plasmid carrying the indicated rec alleles. DNA substrate was derived from pBR322 χ+ and labeled with 32P at the 5'end as described in Materials and Methods. Substrate (4.5 fmol/assay) was incubated with indicated amount of crude extracts and purified RecBCD at 37°C for 2 min in the reaction mixture containing 3.5 mM MgCl2 and 5 mM ATP. The reaction was stopped by addition of EDTA (0.125 M) and SDS (2.5%). Lanes 1 and 2 show the initial dsDNA substrate and the heat-denatured substrate, respectively. The reaction products were monitored on a 1% agarose gel and analyzed by autoradiography. [file 1471-2091-9-27-S1.pdf]

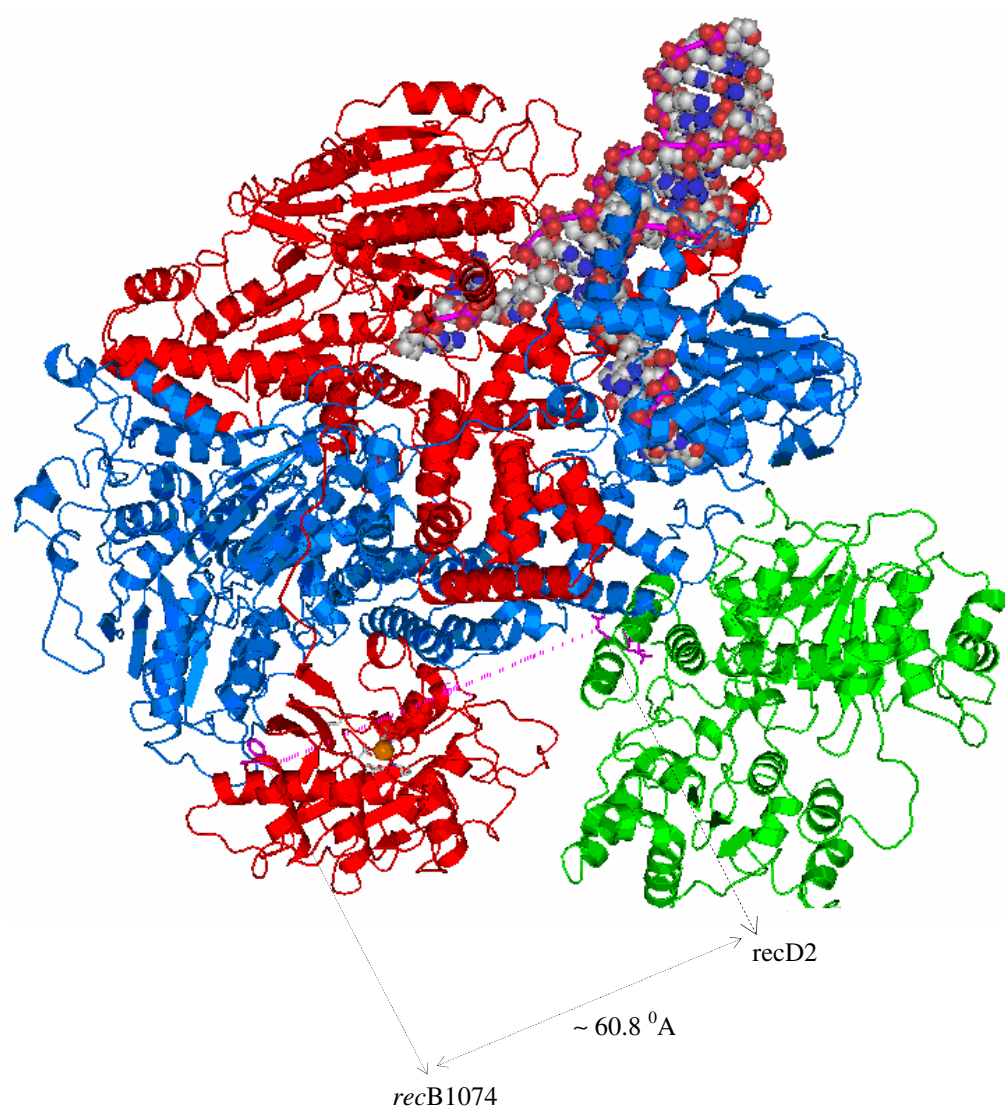

Supplement: Additional File 2 — The distance between C-terminal of RecB and N-terminal of RecD polypeptides. This picture clearly shows the distance between C-terminal of RecB and N-terminal of RecD polypeptides in 3D structure of RecBCD. [file 1471-2091-9-27-S2.pdf]
